# Use of chia (*Salvia hispanica* L.) seed mucilage powder as a stabilizer in the preparation of salep beverage

**DOI:** 10.1002/fsn3.4200

**Published:** 2024-05-15

**Authors:** Alev Altaş, Oguz Gursoy, Hande Özge Güler Dal, Yusuf Yilmaz

**Affiliations:** ^1^ Burdur Directorate of Provincial Agriculture and Forestry, Food and Feed Division Republic of Turkey Ministry of Agriculture and Forestry Burdur Turkey; ^2^ Division of Food Engineering Institute of Natural and Applied Sciences, Burdur Mehmet Akif Ersoy University Burdur Turkey; ^3^ Department of Food Engineering, Faculty of Engineering and Architecture Burdur Mehmet Akif Ersoy University Burdur Turkey

**Keywords:** chia seed, mucilage, orchids, rheology, salep, serum separation, stabilizer

## Abstract

Salep, a traditional Turkish beverage, derives its name from its primary component: salep powder (SP), which is sourced from the tubers of certain orchid species. This study investigated various physicochemical attributes (including dry matter, protein content, pH, titration acidity, water activity, color, serum separation, and zeta potential), as well as rheological and sensory characteristics of salep beverages. These drinks were prepared by substituting SP with chia (*Salvia hispanica* L.) seed mucilage powder (MP) with different ratios (10%, 20%, 30%, and 40%). The substitution of SP with MP did not influence the dry matter and protein contents or the pH and acidity values of the drinks significantly (*p* > .05). The inclusion of MP in the formulation of salep drinks resulted in a decrease in lightness (L*) and a* color values while increasing the b* color values. However, consumer perception, as indicated by color difference values (∆E*), showed no distinguishable difference between drinks containing MP and control drinks. Furthermore, higher ratios of MP led to increased apparent viscosity values in the drinks and effectively prevented or significantly reduced serum separation observed in control drinks (*p* < .05). Remarkably, sensory evaluations revealed that substituting up to 30% of SP with MP did not negatively impact the overall sensory properties of the drinks (*p* > .05), suggesting that MP could be recommended as a feasible alternative. This substitution has the potential to contribute to the conservation of orchid plants, the primary source of salep, while also offering cost‐saving benefits in the production of salep drinks.

## Graphical abstract

The objective was to minimize the reliance on salep powder in the production of salep beverages by incorporating chia seed mucilage as a stabilizer. Chia seed mucilage extracted from chia seeds was used as a substitute for salep powder in varying proportions during the preparation of hot salep drinks. Various physicochemical, rheological, and sensory properties of the salep drinks were determined.

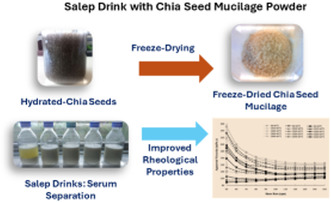

## 1 INTRODUCTION

Salep, derived from the tubers of orchid species, serves as the primary ingredient in the traditional Turkish beverage known as salep drink (Tamer et al., 2006). Typically enjoyed during the winter months, this drink is prepared by boiling SP and sugar in milk, often garnished with a sprinkle of cinnamon. With an increase in demand for salep drinks in recent years, ready‐to‐drink salep drink products have also found their place on store shelves (Doğan & Kayacier, 2004). Consumer preferences for beverages, particularly in the case of hot salep drinks, are greatly influenced by the fluidity properties of beverages. Achieving an optimal consistency in hot salep drinks is crucial for a pleasant mouth feel. The rheological properties of beverages like hot salep or hot chocolate play a significant role in determining product quality (Doğan & Kayacier, 2004; Kayacier & Doğan, 2006).

Salep powder is produced by grinding the tough, dried, light‐yellow tubers of certain perennial orchid species belonging to the *Orchidaceae* family (Tekinşen & Güner, 2010). Orchids, found on every continent except Antarctica, have a remarkable adaptability to diverse altitudes ranging from sea level to 5000 meters, making them the oldest known flowering plants with a history dating back 80 million years (Şen, 2016). Among European countries, Turkey stands out as one of the most biodiverse in terms of orchid species, boasting approximately 170 varieties (Şen, 2017). Presently, Turkey is home to at least 20 endemic orchid species, representing approximately 13% of the total orchid population in the country (Kreutz, 2002). Salep production in Turkey utilizes around 120 orchid species belonging to *Ophrys*, *Orchis*, *Himantoglossum*, *Serapias*, *Anacamptis*, *Comperia*, *Barlia*, *Dactylorhiza*, *Aceras*, *Neotinea*, and other genera growing in various regions of Anatolia (Sezik, 2002). A single dried salep tuber usually weighs between 0.25 and 1.00 g. Consequently, it takes approximately 1000 to 4000 orchid tubers to yield 1 kg of salep. Turkey produces an estimated 45 tons of salep annually, which necessitates the uprooting of an astonishing 45–180 million orchids from the soil. On the other hand, factors such as agricultural expansion, urban development, overgrazing, and deforestation pose significant threats to many rare or even endemic orchid species, placing them at risk of extinction (Kreutz, 2002). The high cost of salep and its extraction from endangered plant species have prompted increased research into natural stabilizers that could serve as alternatives to salep.

Salep powder, a key ingredient in salep drinks, serves as both a thickening and stabilizing agent while giving the desired taste and aroma to the final product. It is also used in the production of Maraş‐type ice cream, where it serves as a thickener and increases the melting point (Tekinşen & Güner, 2010). The thick structure of salep drink, and stiff and unique, slow‐melting characteristic of Maraş‐type ice cream are attributed to the glucomannan content present in salep (Şen, 2016).

*Salvia hispanica* L., commonly known as chia, is an annual plant belonging to the Lamiaceae family, typically thriving in arid or semi‐arid climates (Sandoval‐Oliveros & Paredes‐Lopez, 2013). Chia seeds are renowned for containing the highest known content of ω‐3 linolenic acid among natural sources (Coates & Ayerza, 1996). With their rich fatty acid and protein content, chia seeds can be utilized to produce protein concentrates and ω‐3 capsules, enhancing the nutritional profile of bakery products, cereals, and various foods (Coelho & Salas‐Mellado, 2014). Containing about 50–60 g/kg of mucilage as a soluble dietary fiber, chia seeds possess a unique characteristic: it can easily be extracted and absorb up to 27 times its weight in water when soaked (Capitani et al., 2013). The mucilage, thanks to its unique functional properties (such as structure regulation, emulsification, and thickening), attracts considerable attention in the food industry. Beyond its role as a dietary fiber source, this mucilage polysaccharide is deemed beneficial due to its capacity to lower the glycemic index and induce a sense of satiety (Chiang et al., 2021). Given its technological and physiological attributes, chia mucilage is increasingly recognized as a versatile ingredient. It has been explored as a novel emulsifying agent or thickener in both the cosmetics and food industries, contributing to consumer health as a rich source of soluble dietary fiber (Capitani et al., 2013; Coelho & Salas‐Mellado, 2014; Tas et al., 2023).

In this study, it was aimed to explore the possibility of reducing the reliance on SP in the preparation of salep drinks by using chia seed mucilage as a stabilizer. The ultimate intention was to contribute to the conservation of salep orchids while simultaneously reducing production costs and retail prices of salep drinks. Chia seed mucilage was considered a natural alternative to SP. To achieve this goal, mucilage extracted from soaked chia seeds, obtained through drying and sieving, was used to replace SP at various ratios in the preparation of hot salep drinks. The study investigated various physicochemical, rheological, and sensory properties of the drinks to assess the feasibility and efficacy of this substitution.

## 2 MATERIALS AND METHODS

### 2.1 Materials

Cow's milk (UHT milk with 3.1% fat: Ak Food Industry and Trade Inc., Istanbul, Turkey), chia seeds (Ala Çiftçi, Yayla Agro Food Industry and Transport Inc., Ankara, Turkey), granulated sugar (Konya Sugar Industry and Trade Inc., Konya, Turkey), and salep powder (Aziz Gündüz Nine Brothers Salep and Morel Trade, Burdur, Turkey) used in the preparation of salep drinks were purchased from different local or national grocery stores. Phenolphthalein and NaOH were purchased from Sigma‐Aldrich (St. Louis, MO, USA).

### 2.2 Mucilage extraction

The method of Chaves et al. (2018) was modified for the extraction of chia seed mucilage. Mucilage was extracted from seeds using a water‐to‐seed ratio of 20:1 (v/w). The mixture of seeds and distilled water was heated to 80 ± 2°C and stirred at 750 rpm for 2 h using a hot‐plate magnetic stirrer (WiseStir Wisd, Daihan Scientific Co. Ltd., Gang‐Won‐Do, South Korea). Following this, the aqueous suspension was cooled to room temperature, frozen at −18 ± 1°C, and subsequently subjected to freeze drying for 72 h using a freeze drier (Bluewave, BW‐10B, Bluewave Industry Co. Ltd., Shanghai, China). This freeze‐dried mixture was sieved through a 35‐mesh sieve to separate the mucilage from the seeds. The extracted mucilage was then stored at 4 ± 1°C until needed for the preparation of salep drinks. The quantity of mucilage extracted was determined using a precision balance (A&D Company, Ltd., Tokyo, Japan). The chia mucilage yield (%) was calculated by Equation 1 as described by Feizi et al. (2021).

(1) $$\text{Yield}\%\left(w/w\right)=\frac{\text{Mass of extracted mucilage}\;\left(g\right)}{\text{Mass of chia seeds}\;\left(g\right)}\times 100$$

### 2.3 Preparation of Salep drinks

Salep drinks were prepared using a combination of milk, sugar, and stabilizers, which included SP and/or chia seed MP. The ratios of granulated sugar and the total stabilizer in milk were 5.0 and 0.7% (w/v), respectively. Five different combinations of drinks were prepared, each with varying ratios of chia seed MP and SP. The codes assigned to these drinks were as follows: salep drinks were prepared using a combination of milk, sugar, and stabilizers, which included SP and/or chia seed MP. The ratio of granulated sugar to the total stabilizer in the milk was 5.0% (w/v) and 0.7%, respectively. Five different combinations of drinks were prepared, each with varying ratios of chia seed MP and SP. The codes assigned to these drinks were as follows: KS: control drink with 0.7% SP; CS10: drink with a 10% substitution ratio, consisting of 0.63% SP and 0.07% chia seed MP; CS20: drink with a 20% substitution ratio, consisting of 0.56% SP and 0.14% chia seed MP; CS30: drink with a 30% substitution ratio, consisting of 0.49% SP and 0.21% chia seed MP; and CS40: drink with a 40% substitution ratio, consisting of 0.42% SP and 0.28% chia seed MP.

In the preparation of salep drinks, milk was poured into glass containers with lids and heated to a temperature of 70 ± 2°C using a water bath (WiseBath Wisd, Daihan Scientific Co., Gang‐Won‐Do, South Korea). Granulated sugar and the mixture of stabilizers were then slowly added into the heated milk while being thoroughly mixed. The final drinks were maintained at a constant temperature for 15 min. Following a method similar to Capitani et al. (2012), the salep drinks were homogenized at 7600 rpm for 2 min using a homogenizer (WiseTis HG‐15D, Daihan Scientific Co. Ltd., Gang‐Won‐Do, South Korea). Traditionally, salep drinks are stored for a maximum of 2 days in small enterprises producing traditional salep drinks. Therefore, in this study, the salep drinks were refrigerated at 4 ± 1°C for 2 days.

### 2.4 Physicochemical analyses

The compositional properties (dry matter, fat, lactose, and protein contents) of cow's milk used in the production of the salep drinks were determined using a milk analyzer (Bentley B150, Bentley Instruments Inc., Chaska, Minnesota, USA). The pH values of both milk and salep drinks were determined by a pH meter (Jenco 6173, Jenco, San Diego, CA, USA) while titration acidity values were determined according to the method in Raw Milk Standard (TS 1018) (Anonymous., 2002) by Turkish Standards Institute and Gürsel and Anlı (2018).

The dry matter contents of the salep drinks were determined using a rapid moisture analyzer (Kern DSB, Balingen, Germany) at 105°C. The total nitrogen contents of the drinks were determined according to the Dumas method with a Dumatherm analyzer (Gerhardt GmbH & Co. KG, Königswinter, Germany). The protein contents of the drinks were calculated by multiplying the total nitrogen contents by a conversion factor of 6.38 (Gürsoy et al., 2016). Additionally, the water activity (a_w_) values of the drinks were determined using the water activity device (Testo 645, Testo Inc., Lenzkirch, Germany).

### 2.5 Color measurements

The color values of the drinks were assessed using the CIELAB (Commission Internationale de l'Eclairage) color system, which includes parameters such as L* (lightness), a* (red–green), and b* (yellow–blue). Measurements were conducted using a colorimeter (CR‐400, Konica Minolta, Tokyo, Japan) equipped with a reflectance specular, a D65 illuminator, an observer angle of 10°, and an 8 mm aperture. In the CIELAB system, L* values range from 0 (black) to 100 (white), with positive a* values indicating redness and positive b* values indicating yellowness, while negative values of a* and b* represent greenness and blueness, respectively (Gürsoy et al., 2016).

The whiteness index (WI) for each drink was calculated using the method outlined by Kurt and Atalar (2018). The hue angles (h) were calculated based on the a* and b* color parameters using the method suggested by McLellan et al. (1995). Chroma values (C*) were calculated according to the method proposed by Daraghmah and Qubbaj (2022). Additionally, color difference (∆E*) values were determined using Equation 2, as described by Cecchini et al. (2011).

(2) $$\Delta E=\sqrt{{\left(\Delta L^{*}\right)}^{2}+{\left(\Delta a^{*}\right)}^{2}+{\left(\Delta b^{*}\right)}^{2}}$$

### 2.6 Serum separation

For serum separation, salep drinks were poured into measuring cylinders (25 mL) and stored at 4 ± 1°C. The volume of serum accumulated on the surface was measured on the 1st, 2nd, and 7th days of storage, expressed as serum separation values (%) (Aloğlu, 2018).

### 2.7 Zeta potential

The zeta (ζ) potential values of the drinks were determined using a nanoparticle analyzer (SZ‐100‐Z2, Horiba Scientific, Horiba Limited, Kyoto, Japan) at a temperature of 25°C and an electrode voltage of 2.2 V. Zeta potential (mV) values were calculated based on the electrophoretic mobility of the particles dispersed in the drinks and were recorded accordingly.

### 2.8 Rheological analyses

The rheological properties of salep drinks were determined using a viscometer (DV2T, Brookfield Engineering Laboratories, Middleborough, Massachusetts, USA) equipped with an RV2 spindle. For this purpose, approximately 500 mL of each drink was transferred into beakers insulated by Styrofoam. Measurements were taken at a constant temperature of 40 ± 2, 50 ± 2, and 60 ± 2°C, with rotation speeds ranging from 50 to 150 rpm. For each rotation speed and temperature, torque (%) and viscosity (mPa∙s) values were recorded. Apparent viscosity values of the drinks were determined at a rotation speed of 120 rpm (111.6 s^−1^). The flow behavior index (n) and consistency coefficient (K, Pa∙s^n^) values were calculated using the power law model (Steffe, 1996) in Equation 3, where *δ* and *γ* represent the shear stress (Pa) and the shear rate (s^−1^), respectively.

The Arrhenius equation (Equation 4) was used to calculate the activation energies and the temperature dependence of apparent viscosity values of the salep drinks (Gürsoy et al., 2020). In this equation, *η*, *A*, *E*_*a*_, *R*, and *T* values represent the apparent viscosity (Pa∙s), the frequency factor (Pa∙s), the activation energy (J/mol), the gas constant (8.314 J/mol∙K), and the absolute temperature (*K*) values, respectively (Cuomo et al., 2020).

(3) $$\delta =K{\left(\gamma \right)}^{n}$$

(4) $$\eta =A\;e^{\left(-E_{a}/\mathrm{RT}\right)}$$

### 2.9 Sensory evaluation

The sensory evaluation of the salep drinks was conducted using a 7‐point hedonic scale, where the numerical value of 1 represented “dislike extremely” and 7 indicated “like extremely” (Bodyfelt et al., 1988). The sensory analysis involved 15 untrained (but experienced) panelists who evaluated the drinks based on various attributes such as color, odor, taste, consistency, mouthfeel, and overall liking. Each salep drink, identified with an arbitrary three‐digit code, was presented to the panelists in a randomized order at a temperature of 60 ± 2°C. For the 2nd‐day analyses, the drinks were stored in a refrigerator at 4 ± 1°C and reheated to 60 ± 2°C before presentation to the panelists. Panelists were instructed to use water (Nazlı, Aydın, Turkey) to clean their palate between samples.

The index of acceptability (IA) for the salep drinks was calculated using Equation 5. A food product with an IA of at least 70% is generally considered suitable for consumption (Fernandes & Salas‐Mellado, 2017).

(5) $$\mathrm{IA}\left(\%\right)=\frac{\text{Score}}{7}\times 100$$

### 2.10 Statistical analyses

The analysis of variance (two‐way ANOVA) and Duncan multiple‐comparison test at the significance level of *α* = 0.05 were used by SAS statistical software program (The SAS System for Windows 9.0, SAS Institute Inc., Carry, NC, USA). The results were expressed as mean ± standard deviation. All experiments were conducted in two independent replicates and each analysis was carried out in at least two parallel trials to ensure reliability and consistency of the results.

## 3 RESULTS AND DISCUSSION

### 3.1 Mucilage yield

In this study, a mucilage yield of 8.63 ± 0.50% (w/w) was obtained during mucilage production. Similarly, Chaves et al. (2018) reported a mucilage yield of 8.49% (±0.27) by freeze‐drying chia seeds, using a water‐to‐seed ratio of 40:1 at a pH of 8.0 for 2 h at 80°C. In line with these findings, Muñoz et al. (2012) achieved an optimal yield value of 6.97% through conventional drying of chia seeds under similar conditions. The study by Muñoz et al. (2012) highlighted the significant influence of temperature and seed‐to‐water ratio on chia mucilage yield, emphasizing the importance of optimizing these factors for efficient mucilage extraction. The result of our study is in good agreement with the findings of these previous studies, confirming the consistency and reliability of mucilage yield obtained under optimized extraction conditions.

### 3.2 Physicochemical properties

The cow milk used in the preparation of salep drinks exhibited the following compositional values: dry matter 10.46 ± 0.10%, nonfat dry matter 7.80 ± 0.01%, fat 2.67 ± 0.09%, protein 2.73 ± 0.04%, lactose 4.34 ± 0.01%, pH 6.67 ± 0.03, and titration acidity (expressed in lactic acid %) 0.143 ± 0.00%. Regarding the salep drinks, the dry matter and protein contents were determined in the ranges 18.11–18.32% and 2.83–3.69%, respectively (Table 1). It was found that the substitution of SP with MP did not have a significant effect on the dry matter and protein contents of the salep drinks (*p* > .05).

Chia seed mucilage contains high‐purity polysaccharide units (Timilsena et al., 2016) and salep consists mainly of polysaccharide molecules, such as glucomannan and starch (Tekinşen & Güner, 2010). Additionally, the moisture content of chia mucilage has been reported to range from 5.74% to 8.33% depending on the drying rate (Chavan et al., 2017; Garcia e et al., 2022). Tekinşen and Güner (2010) stated that the moisture and nitrogenous substance content of salep typically fall within the ranges 6–12% and 0.5–1%, respectively. These similarities between the composition of chia mucilage and salep provide support for why the salep drinks in our study exhibited similar dry matter and protein contents, despite the substitution of SP with chia seed MP.

**TABLE 1:** Initial dry matter and protein contents of salep drinks prepared by substituting salep powder (SP) with chia seed mucilage powder (MP) (mean ± standard deviation).

Sample code^a^	Dry matter content (%)^b^	Protein content (%)
KS	18.21 ± 0.84^A^	3.15 ± 0.40^A^
CS10	18.32 ± 0.56^A^	2.83 ± 0.32^A^
CS20	18.12 ± 0.44^A^	3.28 ± 0.22^A^
CS30	18.12 ± 0.47^A^	3.69 ± 0.98^A^
CS40	18.11 ± 0.15^A^	3.15 ± 0.42^A^

^a^ KS (0.7% SP) as control drink, CS10 with a mucilage substitution of 10% (0.63% SP + 0.07% MP), CS20 with a mucilage substitution of 20% (0.56% SP + 0.14% MP), CS30 with a mucilage substitution of 30% (0.49% SP + 0.21% MP), and CS40 with a mucilage substitution of 40% (0.42% SP + 0.28% MP).

^b^ Different superscript letters in a column show significant statistical differences (*p* < .05).

The pH and titration acidity (% lactic acid) values of salep drinks over the storage period are shown in Table 2, with statistically significant differences observed in these values (*p* < .05). However, despite these differences, both the pH and titration acidity values remained within narrow ranges 6.44–6.47% and 0.14–0.16%, respectively. It has been reported that for the sour flavor to become apparent, the acidity level in the milk must exceed 0.18% (Gürsel & Anlı, 2018). Therefore, although the substitution of SP with chia seed MP resulted in some statistical differences in the pH and titration acidity values of the drinks, these differences may have minor significance in practice.

**TABLE 2:** pH and acidity values of salep drinks prepared by substituting salep powder (SP) with chia seed mucilage powder (MP) for two different storage days (mean ± standard deviation).

Sample code^a^	Storage (days)	pH^b^	Acidity (% lactic acid)
KS	0	6.44 ± 0.01^E^	0.142 ± 0.00^CD^
	2	6.49 ± 0.01^BC^	0.147 ± 0.00^BCD^
CS10	0	6.46 ± 0.02^DE^	0.142 ± 0.00^CD^
	2	6.50 ± 0.00^AB^	0.142 ± 0.00^CD^
CS20	0	6.46 ± 0.02^CDE^	0.140 ± 0.00^D^
	2	6.50 ± 0.00^AB^	0.150 ± 0.00^ABC^
CS30	0	6.47 ± 0.02^CD^	0.145 ± 0.00^CD^
	2	6.52 ± 0.02^A^	0.155 ± 0.00^AB^
CS40	0	6.46 ± 0.01^DE^	0.142 ± 0.00^CD^
	2	6.49 ± 0.01^BC^	0.157 ± 0.00^A^

^a^ KS (0.7% SP) as control drink, CS10 with a mucilage substitution of 10% (0.63% SP + 0.07% MP), CS20 with a mucilage substitution of 20% (0.56% SP + 0.14% MP), CS30 with a mucilage substitution of 30% (0.49% SP + 0.21% MP), and CS40 with a mucilage substitution of 40% (0.42% SP + 0.28% MP).

^b^ Different superscript letters in a column show significant statistical differences (*p* < .05).

The water activity values of all salep drinks produced in our study were determined as 0.99. Interestingly, the substitution of SP with chia seed MP did not have a significant effect on the water activity of the drinks (*p* > .05).

### 3.3 Color

Changes in the color values of salep drinks prepared by substituting SP with MP during storage are given in Table 3. Initially, the fresh CS40 drink had a brightness value (L*) of 75.57 ± 0.40 while the KS drink had a brightness value of 77.69 ± 0.77 on its second storage day (*p* < .05). It was found that storing the drinks at 4°C for 2 days did not have a significant effect on the L* color values of salep drinks (*p* > .05). In general, brightness values decreased as the substitution ratio of SP with MP increased, regardless of the storage period (*p* < .05). Campos et al. (2015), in their study on producing ice cream with chia mucilage as a stabilizer, noted a similar trend. The brightness value of the control sample (87.23) decreased to 77.59 with the addition of 1% mucilage and further to 75.81 with 2% mucilage. This decrease was attributed to the slightly creamy color of the mucilage, consistent with our findings.

**TABLE 3:** Color values of salep drinks prepared by substituting salep powder (SP) with chia seed mucilage powder (MP) for two different storage days (mean ± standard deviation).

Sample code^a^	Storage (days)	L*^,^ ^b^	a*	b*	WI	°h	C*
KS	0	77.49 ± 0.37^A^	−2.76 ± 0.33^C^	5.52 ± 0.32^D^	76.66 ± 0.38^A^	116.55 ± 3.38^A^	6.19 ± 0.30^E^
	2	77.69 ± 0.77^A^	−2.73 ± 0.05^C^	6.00 ± 0.31^BC^	76.74 ± 0.72^A^	114.49 ± 1.03^AB^	6.60 ± 0.30^ABC^
CS10	0	76.92 ± 0.46^AB^	−2.47 ± 0.50^ABC^	5.76 ± 0.37^CD^	76.08 ± 0.48^AB^	113.29 ± 5.38^A‐D^	6.29 ± 0.24^DE^
	2	77.00 ± 0.50^AB^	−2.52 ± 0.10^BC^	6.16 ± 0.17^AB^	76.06 ± 0.48^AB^	112.28 ± 0.81^BCD^	6.66 ± 0.18^AB^
CS20	0	75.86 ± 0.39^CD^	−2.61 ± 0.19^BC^	5.77 ± 0.22^CD^	75.05 ± 0.37^CD^	114.35 ± 2.40^AB^	6.35 ± 0.14^CDE^
	2	76.43 ± 0.77^BC^	−2.59 ± 0.07^BC^	5.96 ± 011^BC^	75.55 ± 0.76^BC^	113.54 ± 0.30^ABC^	6.50 ± 0.13^A‐D^
CS30	0	75.79 ± 0.48^CD^	−2.36 ± 0.35^AB^	5.80 ± 0.03^CD^	74.99 ± 0.49^CD^	112.14 ± 3.00^BCD^	6.27 ± 0.14^DE^
	2	76.09 ± 0.64^CD^	−2.27 ± 0.08^AB^	6.26 ± 0.11^AB^	75.18 ± 0.64^CD^	109.94 ± 0.69^DE^	6.67 ± 0.13^AB^
CS40	0	75.57 ± 0.40^D^	−2.28 ± 0.21^AB^	6.02 ± 0.16^BC^	74.74 ± 0.43^D^	110.74 ± 1.47^CDE^	6.45 ± 0.21^B‐E^
	2	75.65 ± 0.63^CD^	−2.14 ± 0.10^A^	6.39 ± 0.05^A^	74.74 ± 0.62^D^	108.48 ± 0.92^E^	6.75 ± 0.06^A^

^a^ KS (0.7% SP) as control drink, CS10 with a mucilage substitution of 10% (0.63% SP + 0.07% MP), CS20 with a mucilage substitution of 20% (0.56% SP + 0.14% MP), CS30 with a mucilage substitution of 30% (0.49% SP + 0.21% MP), and CS40 with a mucilage substitution of 40% (0.42% SP + 0.28% MP).

^b^ Different superscript letters in a column show significant statistical differences (*p* < .05).

The a* color value of fresh KS drinks was −2.76 ± 0.33 while it was −2.14 ± 0.10 for the CS40 sample on its second storage day, and the difference in these values was found significant (*p* < .05). The a* color values of KS, CS10, and CS20 drinks were similar (*p* > .05), whereas CS30 and CS40 drinks showed significant differences in their a* values compared to KS drinks (*p* < .05). All salep drinks displayed negative a* color values, indicating a slight greenish hue on both days of storage. Similar results were reported in studies where chia mucilage was incorporated as a stabilizer in ice cream (Arnak & Tarakçı, 2021), mayonnaise (Fernandes & Salas‐Mellado, 2017), and yogurt (Atik et al., 2020).

The b* color value of fresh KS drink was 5.52 ± 0.32 while it was 6.39 ± 0.05 for CS40 drinks on its second storage day, with a significant difference between these values (*p* < .05). Generally, the b* color values of salep drinks increased with a higher substitution ratio of SP with MP, and this increase was found statistically significant (*p* < .05) only for the fresh CS40 drink. Similarly, storing the drinks at 4°C for 2 days led to a statistically significant increase in the b* color values of all the drinks except for CS20 (*p* < .05). The b* color values of all drinks were positive, indicating a slight yellowish hue, with the highest value observed for the CS40 drink. Our results were similar to those of two studies by Campos et al. (2015) and Arnak and Tarakçı (2021), which used chia mucilage in ice cream production.

Differences in the whiteness index (WI) values of salep drinks revealed that the KS drink had a WI of 76.74 ± 0.72, which decreased to 74.74 ± 0.43 for the CS40 drink with 40% substitution of SP with chia seed MP (*p* < .05). The WI values of KS and CS10 drinks were found similar to those of CS20, CS30, and CS40 drinks (*p* > .05). Storage time did not influence the WI values of salep drinks (*p* > .05). A similar trend was observed for the changes in the L* color values, possibly due to the color of chia MP. The hue angle (h) of the KS drink was 116.55° ± 3.38, and it was 108.48° ± 0.92 for the CS40 drink (*p* < .05). Insignificant changes in the h values of drinks were found between storage days (*p* > .05). The hue angles of fresh KS and CS10 and CS20 drinks were found similar (*p* > .05), whereas those of CS30 and CS40 drinks were statistically different from fresh KS drink (*p* < .05). Strong yellow tones in color were observed as the chia MP ratio increased in salep drinks. Additionally, it was also found that the C* values increased for each drink during storage, resulting in statistically significant differences (*p* < .05) except for CS20. An increase in the C* values of drinks during storage indicated that the color intensity of drinks also increased. This might be due to an increase in the amount of brown pigments in the medium during storage (Ansari & Sahoo, 2018).

The color difference values (∆E*) of salep drinks prepared by substituting SP with MP during storage are given in Table 4. The smallest ∆E* value was 0.68 for the fresh CS10 drink, and the largest was 2.16 for the CS40 drink on its second storage day. According to Cecchini et al. (2011), a ∆E* of 5 is generally considered the threshold where noticeable color differences are detectable by the naked eye. All drinks had ∆E* values below 5, indicating that color differences were scarcely noticed through visual inspection. These results were corroborated by the sensory evaluation data regarding color, as discussed in the subsequent section.

**TABLE 4:** Total color difference (∆E*) values of salep drinks prepared by substituting salep powder (SP) with chia seed mucilage powder (MP) for two different storage days (mean ± standard deviation).

Sample code^a^	Storage (days)	∆E*
CS10	0	0.68
	2	0.74
CS20	0	1.66
	2	1.27
CS30	0	1.77
	2	1.68
CS40	0	2.04
	2	2.16

^a^ KS (0.7% SP) as control drink, CS10 with a mucilage substitution of 10% (0.63% SP + 0.07% MP), CS20 with a mucilage substitution of 20% (0.56% SP + 0.14% MP), CS30 with a mucilage substitution of 30% (0.49% SP + 0.21% MP), and CS40 with a mucilage substitution of 40% (0.42% SP + 0.28% MP).

### 3.4 Serum separation

The serum separation values (%) for KS and CS10 drinks on the 1st, 2nd, and 7th days of storage are given in Table 5. On the 1st day of storage, KS drinks exhibited a serum separation of 37.00%, accompanied by the formation of a cream layer on the surface. This rate increased to 72.50% by the end of the storage period. The occurrence of serum separation and cream formation is linked with the thermodynamic incompatibility between glucomannan and milk proteins in salep (Dai et al., 2017). The serum separation levels of KS drinks remained consistent on the 2nd and 7th days of storage (*p* > .05), and they were statistically different from the value observed on the 1st day of storage (*p* < .05). Conversely, CS10 drinks exhibited a substantial decrease in serum separation compared to KS drinks, attributed to the substitution of SP with chia MP, maintaining a level of 3.00% even after 7 days of storage (*p* < .05). Despite the expected shelf life of salep drinks being 2 days, it was found that drinks with chia seed MP remained stable even after 7 days of storage. For CS20, CS30, and CS40 drinks, serum separation was not observed until the end of storage. The reduction of SP in the preparation of salep drink, achieved by substituting it with chia seed MP, resulted in a significant decrease or complete prevention of serum separation (*p* < .05). This discovery suggests that chia seed MP could be potentially utilized to control or prevent serum separation in liquid foods such as salep drinks.

**TABLE 5:** Serum separation values for salep drinks prepared by substituting salep powder (SP) with chia seed mucilage powder (MP) for three different storage days (mean ± standard deviation).

Sample code^a^	Storage (days)	Serum separation (%)^b^
KS	1	37.00 ± 15.44^B^
	2	68.50 ± 1.91^A^
	7	72.50 ± 1.00^A^
CS10	1	2.50 ± 1.00^C^
	2	3.00 ± 1.15^C^
	7	3.00 ± 1.15^C^

^a^ KS (0.7% SP) as control drink and CS10 with a mucilage substitution of 10% (0.63% SP + 0.07% MP).

^b^ Different superscript letters in a column show significant statistical differences (*p* < .05).

### 3.5 Zeta potential

Zeta potential serves as a crucial indicator for assessing the stability of dispersions, reflecting their ability to maintain stability within a given medium. With the electrical repulsion force being pivotal in preventing colloidal particle aggregation, a higher absolute value of zeta potential typically signifies a more stable structure (Liu et al., 2017). In the case of salep drinks, the mean zeta potential values decreased from −0.40 ± 1.27 mV (KS) to −0.98 ± 1.04 mV (CS40) with increasing the substitution ratio of SP with chia seed MP. The zeta potential values of CS10, CS20, and CS30 drinks were − 0.77 ± 1.20, −0.82 ± 0.91, and − 0.80 ± 0.78, respectively. However, the incorporation of chia seed MP did not yield statistically significant changes in the zeta potential values of the drinks (*p* > .05). These absolute values were quite low and approached neutral compared to findings from various studies on SP polysaccharides (Georgiadis et al., 2012), glucomannan–milk solutions (Dai et al., 2017), and chia mucilage solutions (da Silveira Ramos et al., 2021; Timilsena et al., 2016). Solely considering zeta potential values, it would be expected that salep drinks prepared in this study would be highly susceptible to serum separation. On the other hand, it was found that serum separation in salep drinks containing chia seed MP was either significantly reduced or entirely prevented for up to 7 days. This shows that factors beyond zeta potential may contribute to the stable structure of salep drinks incorporating chia seed MP.

### 3.6 Rheological properties

The rheological properties of fresh salep drinks at three different temperatures (40, 50, and 60°C) are given in Table 6. Among these, the lowest apparent viscosity was 118.35 mPa∙s for KS drinks while the apparent viscosity of CS30 drinks was 144.95 mPa∙s at 40°C (*p* < .05). Similarly, at 50°C, KS drinks exhibited the lowest apparent viscosity of 103.57 mPa∙s, whereas CS30 drinks had an apparent viscosity of 132.03 mPa∙s. At the highest temperature studied, 60°C, all drinks had relatively uniform apparent viscosity values within a narrower range between 99.09 and 113.23 mPa∙s. Except for CS10 drinks, samples containing MP consistently exhibited significantly higher apparent viscosity values across all three temperatures compared to KS samples (*p* < .05). These findings indicate that salep drinks containing chia seed MP were more viscous than KS drinks. Moreover, the consistency coefficient (K) value, initially determined as 1.70 Pa∙s^n^ in KS drinks, progressively increased with the inclusion of chia seed MP, reaching 7.60 Pa∙s^n^ in CS40 drinks (*p* < .05). Notably, the consistency coefficient value for CS40 drinks was approximately six times greater than that of KS drinks at 50°C (see Table 6). It is worth noting that for data to be deemed reliable, the coefficient of determination (*R* ^2^) should ideally be at least 0.80 (Koocheki et al., 2009). In our study, all *R* ^2^ values obtained from rheological measurements fell within the range of 0.82–1.00, indicating that the power law model was appropriate to explain the relationship among variables, thus ensuring highly dependable results.

**TABLE 6:** Rheological properties of fresh salep drinks prepared by substituting salep powder (SP) with chia seed mucilage powder (MP) at 40, 50, and 60°C (mean ± standard deviation).

Sample code^a^	Temperature (°C)	Apparent viscosity^b^ (120 rpm, mPa∙s)	Consistency coefficient (*K*, Pa∙s^n^)	Flow behavior index (*n*)	*R* ^2^
KS	40	118.35 ± 3.11^EF^	1.70 ± 0.19^G^	1.02 ± 0.04^B^	0.99 ± 0.00
	50	103.57 ± 3.19^G^	1.17 ± 0.06^G^	1.13 ± 0.01^A^	1.00 ± 0.00
	60	100.07 ± 11.32^G^	1.07 ± 0.27^G^	1.17 ± 0.06^A^	1.00 ± 0.00
CS10	40	136.07 ± 1.88^BC^	3.38 ± 1.01^E^	0.81 ± 0.12^DE^	0.98 ± 0.01
	50	112.83 ± 3.21^F^	2.20 ± 0.36^FG^	0.90 ± 0.09^CD^	0.98 ± 0.01
	60	99.09 ± 3.24^G^	1.60 ± 0.28^G^	0.97 ± 0.08^BC^	0.99 ± 0.00
CS20	40	142.00 ± 1.77^AB^	4.87 ± 0.33^CD^	0.67 ± 0.02^FG^	0.94 ± 0.01
	50	127.10 ± 1.38^D^	3.72 ± 0.91^DE^	0.74 ± 0.11^EF^	0.96 ± 0.02
	60	112.43 ± 5.46^F^	3.04 ± 0.23^EF^	0.76 ± 0.02^EF^	0.95 ± 0.00
CS30	40	144.95 ± 1.71^A^	6.80 ± 0.95^AB^	0.55 ± 0.05^H^	0.88 ± 0.03
	50	132.03 ± 2.01^CD^	6.72 ± 0.80^AB^	0.53 ± 0.04^H^	0.86 ± 0.04
	60	113.23 ± 6.70^F^	4.80 ± 0.89^CD^	0.59 ± 0.08^GH^	0.87 ± 0.06
CS40	40	139.65 ± 8.40^ABC^	7.60 ± 1.37^A^	0.50 ± 0.08^H^	0.84 ± 0.04
	50	126.75 ± 8.25^DE^	6.89 ± 1.39^A^	0.50 ± 0.08^H^	0.82 ± 0.08
	60	113.08 ± 11.62^F^	5.68 ± 1.22^BC^	0.53 ± 0.10^H^	0.85 ± 0.05

^a^ KS (0.7% SP) as control drink, CS10 with a mucilage substitution of 10% (0.63% SP + 0.07% MP), CS20 with a mucilage substitution of 20% (0.56% SP + 0.14% MP), CS30 with a mucilage substitution of 30% (0.49% SP + 0.21% MP), and CS40 with a mucilage substitution of 40% (0.42% SP + 0.28% MP).

^b^ Different superscript letters in a column show significant statistical differences (*p* < .05).

The flow behavior index (n) values of KS drinks were 1.02, 1.13, and 1.17 while those for CS40 drinks were 0.50, 0.50, and 0.53 at 40, 50, and 60°C, respectively. The flow behavior index of salep drinks had similar statistical relationships at all three temperatures studied, and these values decreased as the chia seed MP content of drinks increased (*p* < .05).

The rheological properties of salep drinks stored at 4°C for 2 days are given at 40, 50, and 60°C in Table 7. The correlation of variables observed at the beginning of storage largely persisted, with increasing statistical similarities. Notably, CS30 and CS40 drinks had higher apparent viscosity values than the control (KS) group (*p* < .05). Similarly, the consistency coefficient values of the drinks, akin to their apparent viscosity values, decreased after the 2‐day storage period, dropping notably below the values recorded on the production day across all three temperatures. Consistent with the production day observations, higher levels of chia seed MP led to higher consistency coefficient values. Regarding flow behavior index values, differences were statistically insignificant across different temperatures for each sample (*p* > .05). These values generally decreased as the content of chia seed MP increased, aligning with the anticipated behavior observed with increasing apparent viscosity and consistency coefficient values. Similar to the production day, the lowest flow behavior index was determined for CS40 drinks, which had the highest content of chia seed MP (*p* < .05). Generally, an increase in the chia seed MP ratio increased the apparent viscosity and consistency coefficient values of the drinks while reducing their flow behavior index values, indicating a more pronounced shear‐thinning property as the chia seed MP content increased. This phenomenon was attributed to the high purity of chia seed MP, and the reduction of impurities derived from salep as the ratio of SP in formula decreased.

**TABLE 7:** Rheological properties of salep drinks prepared by substituting salep powder (SP) with chia seed mucilage powder (MP) at different temperatures after 2 days of storage (mean ± standard deviation).

Sample code^a^	Temperature (°C)	Apparent viscosity^b^ (120 rpm, mPa∙s)	Consistency coefficient (*K*, Pa∙s^n^)	Flow behavior index (*n*)	*R* ^2^
KS	40	71.25 ± 13.62^D‐G^	0.61 ± 0.16^E^	1.27 ± 0.04^AB^	1.00 ± 0.00
	50	64.08 ± 13.27^EFG^	0.53 ± 0.16^E^	1.30 ± 0.05^AB^	1.00 ± 0.00
	60	51.99 ± 11.42^G^	0.39 ± 0.11^E^	1.34 ± 0.03^A^	1.00 ± 0.00
CS10	40	77.42 ± 14.20^C‐F^	1.08 ± 0.35^DE^	1.05 ± 0.07^CD^	0.99 ± 0.00
	50	68.42 ± 11.48^D‐G^	0.84 ± 0.19^DE^	1.10 ± 0.06^CD^	1.00 ± 0.00
	60	59.58 ± 11.79^FG^	0.62 ± 0.21^E^	1.19 ± 0.07^BC^	1.00 ± 0.00
CS20	40	87.41 ± 16.48^A‐D^	1.70 ± 0.55^CD^	0.91 ± 0.06^EF^	0.98 ± 0.00
	50	75.42 ± 15.71^C‐F^	1.25 ± 0.55^DE^	0.99 ± 0.13^DE^	0.99 ± 0.00
	60	64.58 ± 12.70^EFG^	0.96 ± 0.40^DE^	1.03 ± 0.11^DE^	0.99 ± 0.00
CS30	40	99.84 ± 23.33^AB^	2.72 ± 1.47^BC^	0.79 ± 0.14^F^	0.96 ± 0.01
	50	86.16 ± 16.97^A‐D^	2.32 ± 1.19^C^	0.80 ± 0.14^F^	0.96 ± 0.02
	60	75.17 ± 14.70^C‐F^	1.79 ± 0.82^CD^	0.85 ± 0.17^F^	0.96 ± 0.02
CS40	40	102.67 ± 3.59^A^	4.05 ± 1.15^A^	0.62 ± 0.10^G^	0.89 ± 0.02
	50	91.50 ± 8.89^ABC^	3.42 ± 0.97^AB^	0.65 ± 0.10^G^	0.90 ± 0.04
	60	82.58 ± 5.85^B‐E^	3.45 ± 0.60^AB^	0.60 ± 0.06^G^	0.87 ± 0.01

^a^ KS (0.7% SP) as control drink, CS10 with a mucilage substitution of 10% (0.63% SP + 0.07% MP), CS20 with a mucilage substitution of 20% (0.56% SP + 0.14% MP), CS30 with a mucilage substitution of 30% (0.49% SP + 0.21% MP), and CS40 with a mucilage substitution of 40% (0.42% SP + 0.28% MP).

^b^ Different superscript letters in a column show significant statistical differences (*p* < .05).

The apparent viscosity and consistency coefficient values of all salep drinks decreased with an increase in temperature just like the flow behavior index values of the drinks. This increase in the flow behavior index values of drinks might suggest the weakening network structure of glucomannan in SP, which unfolds under the influence of higher temperatures (Yaşar et al., 2009). Similarly, higher flow behavior index values in beverages with higher chia seed MP contents, which maintained better stability during heating, could imply that mucilage contributes to strengthened molecular forces, preserving viscosity at higher temperatures (Coorey et al., 2014). Salep drinks with increased chia seed MP contents, which had high viscosities and exhibited improved stability at high temperatures, showed the potential for chia seed MP as a thickener in hot beverages like salep. Regarding storage effects, all beverages experienced a decrease in apparent viscosity and consistency coefficient values, alongside an increase in flow behavior index values over time. This suggests potential degradation of chia seed mucilage over time, leading to unstable suspensions (Cuomo et al., 2020).

**FIGURE 1:**
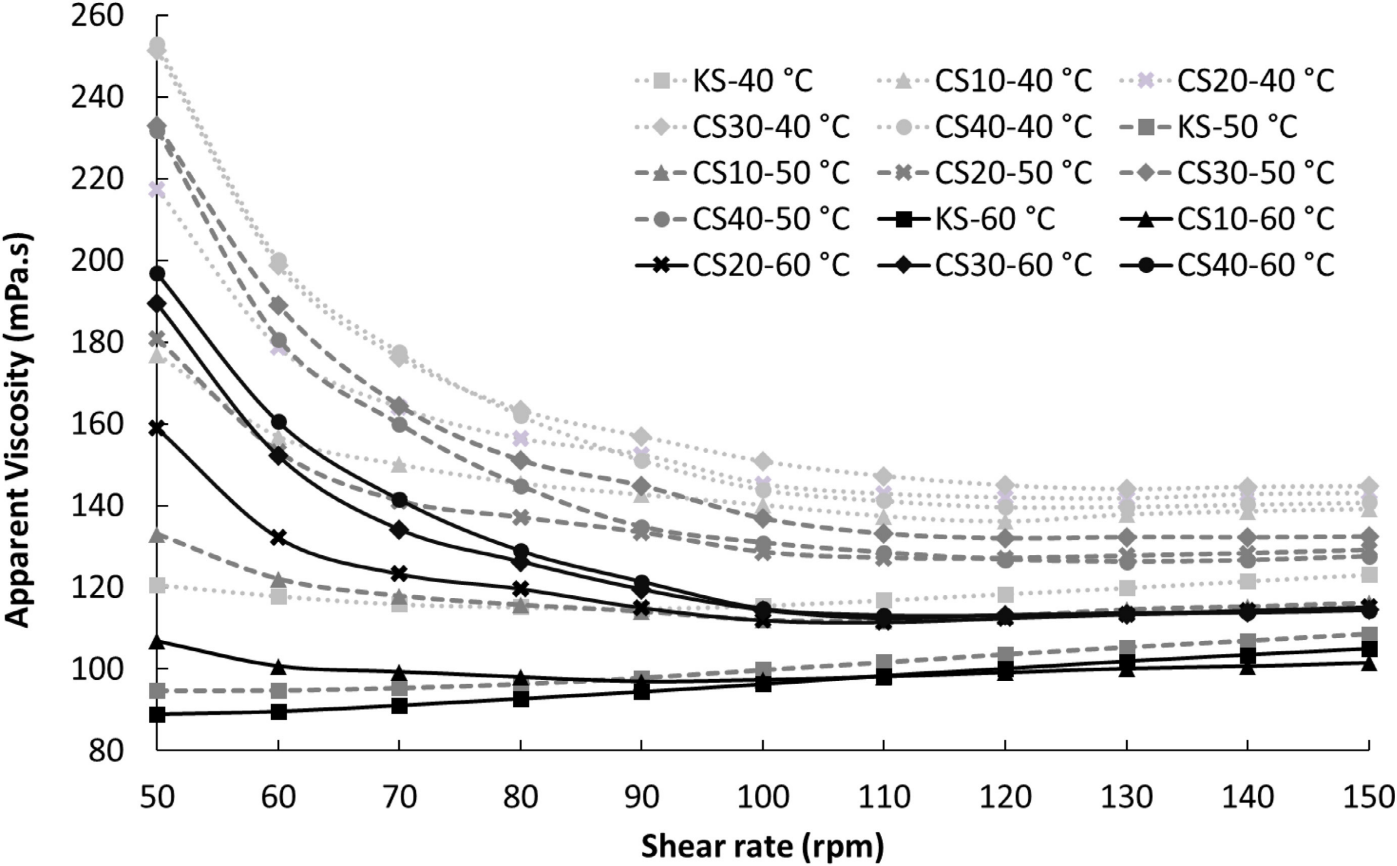
Apparent viscosities of salep drinks prepared by substituting salep powder (SP) with chia seed mucilage powder (MP) on the production day (KS (0.7% SP) as control drink, CS10 with a mucilage substitution of 10% (0.63% SP + 0.07% MP), CS20 with a mucilage substitution of 20% (0.56% SP + 0.14% MP), CS30 with a mucilage substitution of 30% (0.49% SP + 0.21% MP), and CS40 with a mucilage substitution of 40% (0.42% SP + 0.28% MP)).

**FIGURE 2:**
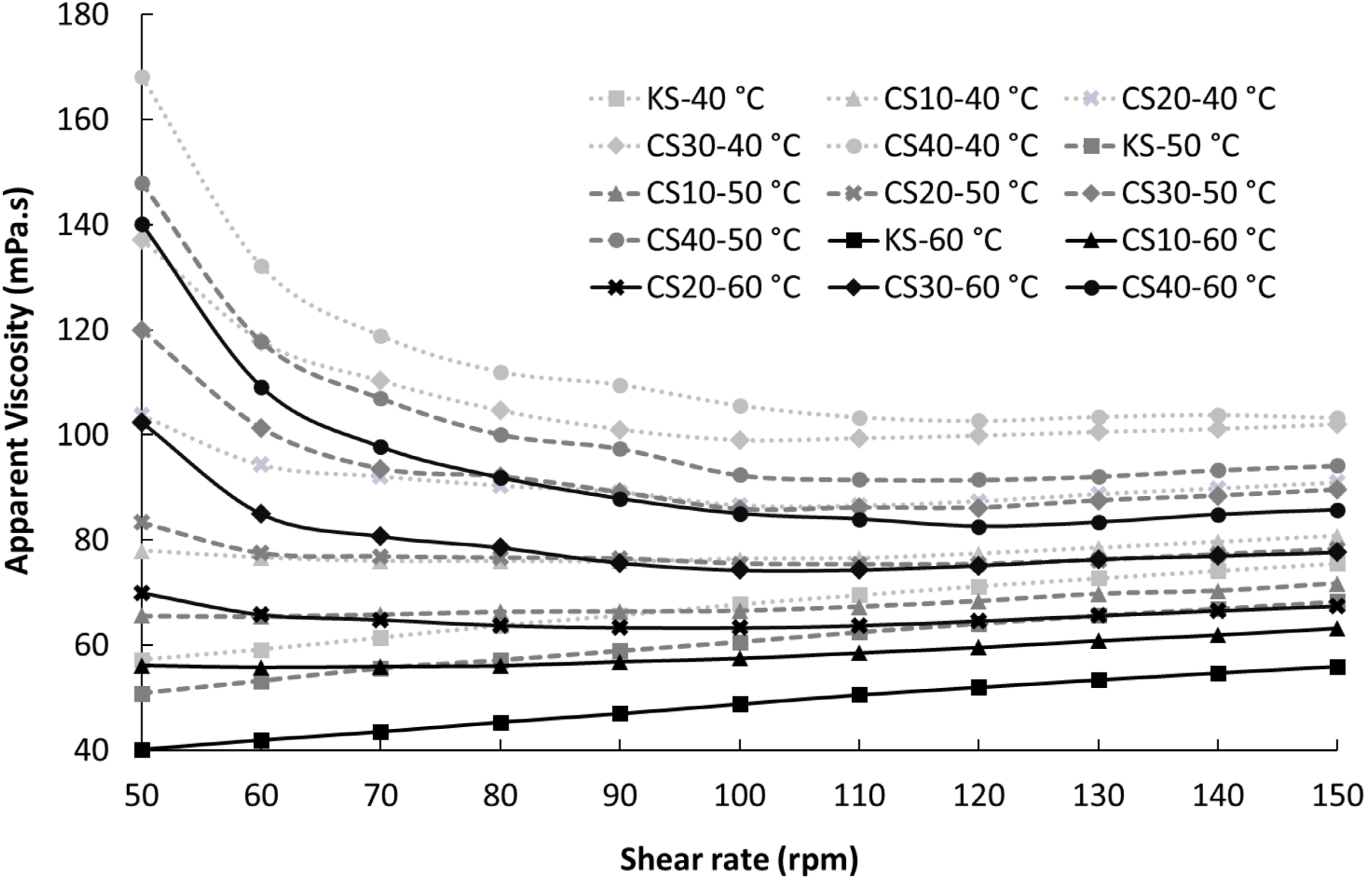
Apparent viscosities of salep drinks prepared by substituting salep powder (SP) with chia seed mucilage powder (MP) after 2 days of storage (KS (0.7% SP) as control drink, CS10 with a mucilage substitution of 10% (0.63% SP + 0.07% MP), CS20 with a mucilage substitution of 20% (0.56% SP + 0.14% MP), CS30 with a mucilage substitution of 30% (0.49% SP + 0.21% MP), and CS40 with a mucilage substitution of 40% (0.42% SP + 0.28% MP)).

Figure 1 displays the apparent viscosity values of salep drinks at various shear rates on the production day, while Figure 2 illustrates the same values for 2 days of storage. It was observed that the apparent viscosity values of salep drinks containing chia seed MP generally decreased with increasing shear rates. This phenomenon can be attributed to the alignment of randomly positioned polymer molecule chains in the flow direction at high shear rates, leading to a decrease in interaction between neighboring polymer chains and subsequently reducing resistance to flow, resulting in decreased viscosity values (Koocheki et al., 2013). Such behavior, characterized by a decrease in apparent viscosity with increasing shear rates, is known as pseudoplastic behavior, indicative of shear thinning. Shear thinning is typically desirable in food products as it contributes to an acceptable mouthfeel by facilitating easier swallowing as viscosity decreases under the tongue's revolving motion. Moreover, shear thinning offers operational advantages in production lines utilizing high‐shear rate processes, facilitating smooth operations such as pumping and filling (Koocheki et al., 2013; Szczesniak & Farkas, 1962).

**FIGURE 3:**
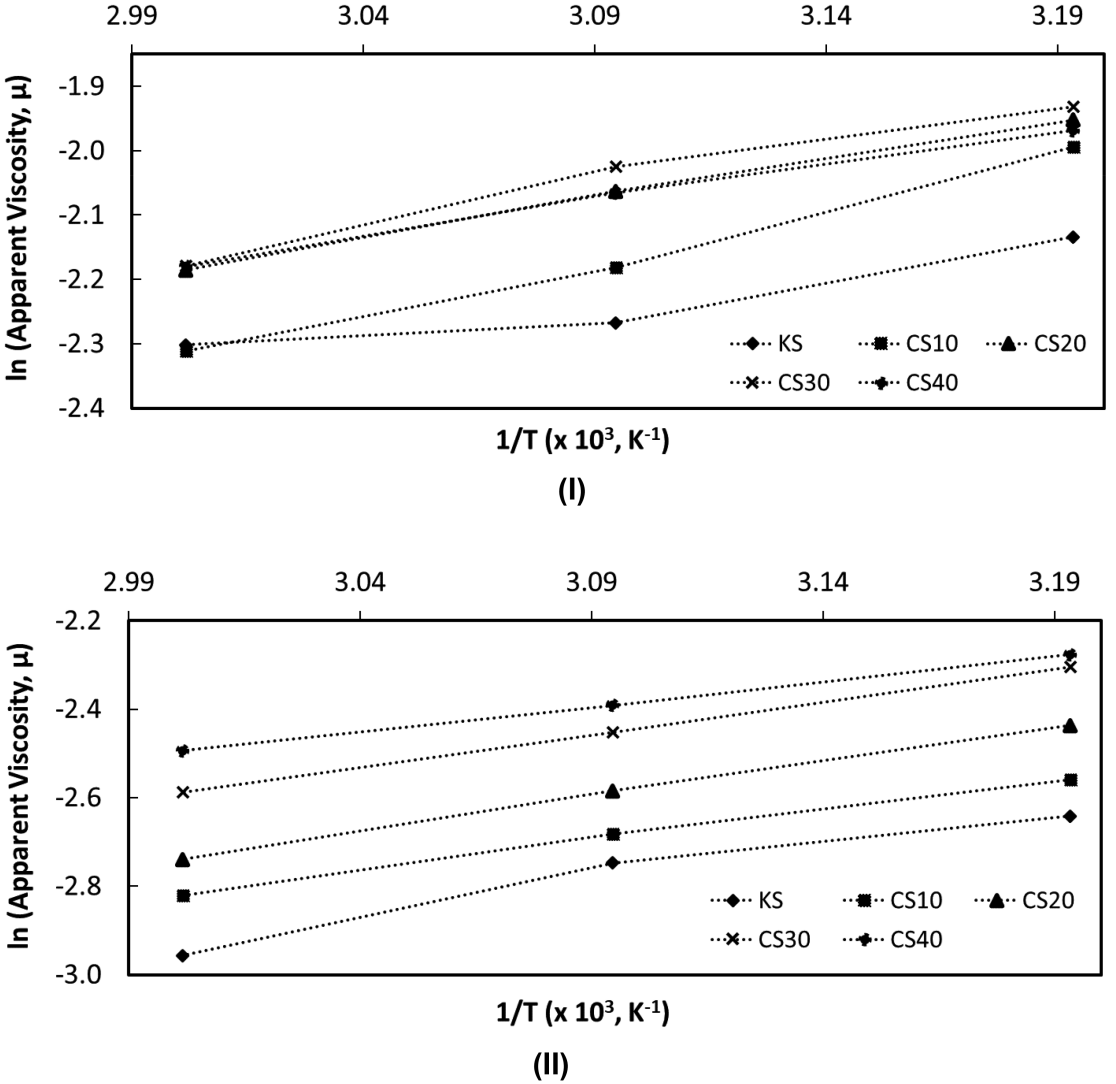
Changes in the apparent viscosities of salep drinks prepared by substituting salep powder (SP) with chia seed mucilage powder (MP) in relation to temperature at production day (I), and after 2 days of storage (II) (KS (0.7% SP) as control drink, CS10 with a mucilage substitution of 10% (0.63% SP + 0.07% MP), CS20 with a mucilage substitution of 20% (0.56% SP + 0.14% MP), CS30 with a mucilage substitution of 30% (0.49% SP + 0.21% MP), and CS40 with a mucilage substitution of 40% (0.42% SP + 0.28% MP)).

The activation energy and frequency factor values of salep drinks calculated using the Arrhenius equation (Equation 4) are given in Table 8. The variation of the apparent viscosities of salep drinks with temperature is shown in Figure 3. It was determined that the *E*_*a*_ value of CS10 drinks (7.01 kJ/mol) exceeded that of the control sample (20.03 kJ/mol), indicating high sensitivity to temperature changes (Cemeroğlu, 2013). Conversely, as the chia seed MP content increased, the *E*_*a*_ values of other drinks decreased, suggesting that chia seed MP helped mitigate viscosity losses with increasing temperatures. Frequency factor values in Table 8 showed that the lowest value at the beginning of the storage period for CS10 drinks (1.29 × 10^−5^ Pa∙s), and the highest for CS40 drinks (6.12 × 10^−2^ Pa∙s), which had the highest chia seed MP content. The increase in frequency factor values with MP content suggests increased collision frequency between reactant molecules and more approaches to the activation barrier, accompanied by reduced mobility of polysaccharide chains (Cemeroğlu, 2015; Cuomo et al., 2020). This corresponds to the expected reduction in impurities as chia seed MP content increases.

**TABLE 8:** Activation energy and frequency factor values of salep drinks prepared by substituting salep powder (SP) with chia seed mucilage powder (MP) for two different storage days determined by using the Arrhenius equation.

Sample code^a^	Storage (days)	Activation energy (*E*_*a*_, kJ/mol)	Frequency factor (A, Pa∙s)
KS	0	20.03	7.39 × 10^−4^
	2	19.06	4.13 × 10^−4^
CS10	0	32.46	1.29 × 10^−5^
	2	24.45	9.13 × 10^−5^
CS20	0	20.49	1.84 × 10^−3^
	2	24.87	1.20 × 10^−4^
CS30	0	14.98	2.27 × 10^−2^
	2	18.03	2.71 × 10^−3^
CS40	0	12.59	6.12 × 10^−2^
	2	7.01	2.70 × 10^−1^

^a^ KS (0.7% SP) as control drink, CS10 with a mucilage substitution of 10% (0.63% SP + 0.07% MP), CS20 with a mucilage substitution of 20% (0.56% SP + 0.14% MP), CS30 with a mucilage substitution of 30% (0.49% SP + 0.21% MP), and CS40 with a mucilage substitution of 40% (0.42% SP + 0.28% MP).

### 3.7 Sensory properties

The sensory attributes including color, odor, taste, consistency, mouthfeel, and overall liking characteristics of salep drinks are given in Table 9 while the index of acceptability values (%) calculated based on these sensory properties are in Table 10. The sensory color scores of salep drinks (Table 9) ranged from 5.46 to 6.13, and the differences in color scores among samples and between storage days were found statistically insignificant (*p* > .05). None of the color scores fell below the 70% limit of index of acceptability (Table 10), suggesting that the effect of the chia seed MP on the CIE color parameters was minimal. Sensory odor scores of all salep drinks with chia seed MP were found similar to the control group (KS) (*p* > .05) at the beginning of the storage period, indicating that the incorporation of chia seed MP did not adversely affect the odor scores of fresh salep drinks. Panelists' reports on sensory evaluation forms indicated that the control drink KS was noted for having an intense salep odor, and the CS10 drink was perceived to have a more pleasant odor and taste. However, after 2 days of storage, the odor scores of salep drinks declined, with the lowest score for KS (4.84 ± 1.81) and the highest for CS30 (5.78 ± 0.94) (*p* < .05). The sensory odor characteristic of KS drink fell below the acceptable limit of 70% after 2 days of storage, indicating that substituting SP with chia seed MP helped maintain the acceptability of drinks in terms of odor. It was determined that the sensory taste scores of all salep drinks, except CS40, were statistically similar at the beginning of the storage period, and were found statistically similar (*p* > .05) as were mouthfeel and overall liking characteristics. The same was also true for the mouthfeel and overall liking characteristics of salep drinks. The CS40 drink on its production day was liked less than the others, with a mouthfeel score of 3.76 ± 1.86, and an overall liking score of 4.23 ± 1.65. Additionally, the IA scores of fresh CS40 drinks fell below the 70% limit for the taste, consistency, mouthfeel, and overall liking characteristics (Table 10). On the other hand, chia seed MP substitution did not significantly influence the sensory consistency scores of drinks (*p* > .05). Control drinks also fell below 70% in terms of consistency by the end of the storage period, similar to the odor characteristic. These results suggest that chia seed MP could replace SP up to 30% in the preparation of salep drinks without adversely impacting sensory liking scores, but substitution rates higher than this may negatively influence consumer preferences for salep drinks.

**TABLE 9:** Sensory properties of salep drinks prepared by substituting salep powder (SP) with chia seed mucilage powder (MP) for two different storage days (mean ± standard deviation).

Sample code^a^	Storage (days)	Color^b^	Odor	Taste	Consistency	Mouthfeel	Overall liking
KS	0	6.03 ± 0.96^A^	5.43 ± 1.71^ABC^	5.56 ± 1.40^A^	5.36 ± 1.49^AB^	5.56 ± 1.45^A^	5.40 ± 1.56^A^
	2	5.46 ± 1.60^A^	4.84 ± 1.81^C^	5.21 ± 1.89^A^	4.75 ± 1.98^B^	5.37 ± 1.79^A^	5.03 ± 1.73^A^
CS10	0	6.13 ± 1.27^A^	6.06 ± 1.01^A^	5.73 ± 1.46^A^	5.70 ± 1.46^A^	5.76 ± 1.61^A^	5.76 ± 1.22^A^
	2	5.65 ± 1.61^A^	5.21 ± 1.47^BC^	5.40 ± 1.75^A^	5.25 ± 1.95^AB^	5.59 ± 1.62^A^	5.53 ± 1.60^A^
CS20	0	5.76 ± 1.33^A^	5.53 ± 0.89^ABC^	5.23 ± 1.40^A^	5.46 ± 1.63^AB^	5.43 ± 1.25^A^	5.26 ± 1.33^A^
	2	5.62 ± 1.68^A^	5.34 ± 1.49^BC^	5.37 ± 1.77^A^	5.53 ± 1.88^AB^	5.43 ± 1.68^A^	5.43 ± 1.68^A^
CS30	0	5.96 ± 1.12^A^	5.43 ± 1.19^ABC^	5.10 ± 1.26^A^	5.06 ± 2.06^AB^	5.23 ± 1.47^A^	5.16 ± 1.34^A^
	2	6.00 ± 1.01^A^	5.78 ± 0.94^AB^	5.43 ± 1.43^A^	5.40 ± 1.68^AB^	5.43 ± 1.64^A^	5.56 ± 1.34^A^
CS40	0	5.56 ± 1.54^A^	5.16 ± 1.46^BC^	3.93 ± 1.92^B^	4.83 ± 1.76^AB^	3.76 ± 1.86^B^	4.23 ± 1.65^B^
	2	5.87 ± 1.15^A^	5.03 ± 1.69^C^	5.15 ± 1.50^A^	5.62 ± 1.43^A^	5.31 ± 1.53^A^	5.21 ± 1.26^A^

^a^ KS (0.7% SP) as control drink, CS10 with a mucilage substitution of 10% (0.63% SP + 0.07% MP), CS20 with a mucilage substitution of 20% (0.56% SP + 0.14% MP), CS30 with a mucilage substitution of 30% (0.49% SP + 0.21% MP), and CS40 with a mucilage substitution of 40% (0.42% SP + 0.28% MP).

^b^ Different superscript letters in a column show significant statistical differences (*p* < .05).

**TABLE 10:** Index of acceptability rates (%) for the sensory properties of salep drinks prepared by substituting salep powder (SP) with chia seed mucilage powder (MP) for two different storage days.

Sample code^a^	Storage (days)	Color (IA)	Odor^b^ (IA)	Taste (IA)	Consistency (IA)	Mouthfeel (IA)	Overall Liking (IA)
KS	0	86.14	77.57	79.43	76.57	79.43	77.14
	2	78.00	**69.14**	74.43	**67.86**	76.71	71.86
CS10	0	87.57	86.57	81.86	81.43	82.29	82.29
	2	80.71	74.43	77.14	75.00	79.86	79.00
CS20	0	82.29	79.00	74.71	78.00	77.57	75.14
	2	80.29	76.29	76.71	79.00	77.57	77.57
CS30	0	85.14	77.57	72.86	72.29	74.71	73.71
	2	85.71	82.57	77.57	77.14	77.57	79.43
CS40	0	79.43	73.71	**56.14**	**69.00**	**53.71**	**60.43**
	2	83.86	71.86	73.57	80.29	75.86	74.43

^a^ KS (0.7% SP) as control drink, CS10 with a mucilage substitution of 10% (0.63% SP + 0.07% MP), CS20 with a mucilage substitution of 20% (0.56% SP + 0.14% MP), CS30 with a mucilage substitution of 30% (0.49% SP + 0.21% MP), and CS40 with a mucilage substitution of 40% (0.42% SP + 0.28% MP).

^b^ IA values lower than 70% were identified in bold.

## 4 CONCLUSION

The substitution of SP with chia seed MP in the preparation of salep drinks did not influence the overall chemical composition of the drinks. Although the CIE color parameters of the salep drinks were influenced, the differences were within levels where consumers could scarcely distinguish the color differences (ΔE* < 5). In general, with an increase in the ratio of chia seed MP, the lightness and greenness of the drinks decreased, while the yellowness increased. The incorporation of chia seed MP into salep drinks also significantly or completely inhibited serum separation in the drinks, a phenomenon observed in control drinks. Furthermore, the rheological properties of the drinks improved as the chia seed MP content of drinks increased. SP could be replaced by chia seed MP up to 30% without adversely affecting the sensory properties of the salep drinks as indicated by the index of acceptability values. In conclusion, chia seed mucilage could serve as a viable alternative stabilizer in substituting SP in the preparation of hot salep drinks, without the need for additional food additives. This substitution also reduces the reliance on SP, derived solely from orchid plants, thereby conserving these plants and reducing production costs for such drinks.

## AUTHOR CONTRIBUTIONS

**Alev Altaş:** Conceptualization (equal); formal analysis (lead); investigation (lead); methodology (equal); writing – original draft (equal); writing – review and editing (equal). **Oguz Gursoy:** Conceptualization (lead); investigation (lead); methodology (lead); resources (lead); supervision (lead); writing – review and editing (lead). **Hande Özge Güler Dal:** Conceptualization (equal); formal analysis (supporting); investigation (supporting); writing – review and editing (equal). **Yusuf Yilmaz:** Conceptualization (equal); formal analysis (supporting); investigation (supporting); methodology (supporting); writing – review and editing (equal).

## CONFLICT OF INTEREST STATEMENT

The authors confirm no conflict of interest with respect to the work described in this manuscript.

## ETHICS STATEMENT

The conducted research is not related to either human or animal use.

## Data Availability

The datasets generated during and/or analyzed during the current study are available from the corresponding author on a reasonable request.

## References

[fsn34200-bib-0001] Aloğlu, H. Ş. (2018). Ayran Analizleri. In Z. Öner & H. Şanlıdere Aloğlu (Eds.), Süt ve Süt Ürünleri Analiz Yöntemleri (pp. 223–224). Sidas Medya.

[fsn34200-bib-0002] Anonymous . (2002). TS 1018 Raw Milk Standard. Turkish Standards Institute.

[fsn34200-bib-0003] Ansari, I. A. , & Sahoo, P. K. (2018). Color variation of ultra high temperature (UHT) sterilized milk during storage. International Journal of Chemical Studies, 6(2), 754–757.

[fsn34200-bib-0004] Arnak, B. G. , & Tarakçı, Z. (2021). Use of chia (*Salvia hispanica* L.) mucilage powder as a replacer of salep in ice cream production. Journal of Food Processing and Preservation, 45(12), e16060. 10.1111/jfpp.16060

[fsn34200-bib-0005] Atik, D. S. , Demirci, T. , Öztürk, H. İ. , Demirci, S. , Sert, D. , & Akın, N. (2020). Chia seed mucilage versus guar gum: Effects on microstructural, textural, and antioxidative properties of set‐type yoghurts. Brazilian Archives of Biology and Technology, 63, e20190702. 10.1590/1678-4324-2020190702

[fsn34200-bib-0006] Bodyfelt, F. W. , Tobias, J. , & Trout, G. M. (1988). The sensory evaluation of dairy products. New York, 598, pp. 227–299.

[fsn34200-bib-0007] Campos, B. E. , Dias Ruivo, T. , da Silva Scapim, M. R. , Madrona, G. S. , & Bergamasco, R. (2015). Optimization of the mucilage extraction process from chia seeds and application in ice cream as a stabilizer and emulsifier. LWT – Food Science and Technology, 65, 874–883. 10.1016/j.lwt.2015.09.021

[fsn34200-bib-0008] Capitani, M. I. , Ixtaina, V. Y. , Nolasco, S. M. , & Tomás, M. C. (2013). Microstructure, chemical composition and mucilage exudation of chia (*Salvia hispanica* L.) nutlets from Argentina. Journal of the Science of Food and Agriculture, 93(15), 3856–3862. 10.1002/jsfa.6327 23900918

[fsn34200-bib-0009] Capitani, M. I. , Spotorno, V. , Nolasco, S. M. , & Tomás, M. C. (2012). Physicochemical and functional characterization of by‐products from chia (*Salvia hispanica* L.) seeds of Argentina. Food Science and Technology, 45, 94–102. 10.1016/j.lwt.2011.07.012

[fsn34200-bib-0010] Cecchini, M. , Contini, M. , Massantini, R. , Monarca, D. , & Moscetti, R. (2011). Effects of controlled atmospheres and low temperature on storability of chestnuts manually and mechanically harvested. Postharvest Biology and Technology, 61, 131–136. 10.1016/j.postharvbio.2011.03.001

[fsn34200-bib-0011] Cemeroğlu, B. S. (2013). Gıda Mühendisliğinde Temel İşlemler. Bizim Grup Basımevi.

[fsn34200-bib-0012] Cemeroğlu, B. S. (2015). Reaksiyon Kinetiği (Gıdaların Bozulma Kinetiği). Bizim Büro Yayınları.

[fsn34200-bib-0013] Chavan, V. R. , Gadhe, K. S. , Dipak, S. , & Hingade, S. T. (2017). Studies on extraction and utilization of chia seed gel in ice cream as a stabilizer. Journal of Pharmacognosy and Phytochemistry, 6(5), 1367–1370.

[fsn34200-bib-0014] Chaves, M. A. , Piati, J. , Malacarne, L. T. , Gall, R. E. , Colla, E. , Bittencourt, P. R. S. , de Souza, A. H. P. , Gomes, S. T. M. , & Matsushita, M. (2018). Extraction and application of chia mucilage (*Salvia hispanica* L.) and locust bean gum (*Ceratonia siliqua* L.) in goat milk frozen dessert. Journal of Food Science and Technology, 55(10), 4148–4158. 10.1007/s13197-018-3344-2 30228413 PMC6133828

[fsn34200-bib-0015] Chiang, J. H. , Ong, D. S. M. , Ng, F. S. K. , Hua, X. Y. , Tay, W. L. W. , & Henry, C. J. (2021). Application of chia (*Salvia hispanica*) mucilage as an ingredient replacer in foods. Trends in Food Science & Technology, 115, 105–116. 10.1016/j.tifs.2021.06.039

[fsn34200-bib-0016] Coates, W. , & Ayerza, R. (1996). Production potential of chia in northwestern Argentina. Industrial Crops and Products, 5, 229–233. 10.1016/0926-6690(96)89454-4

[fsn34200-bib-0017] Coelho, M. S. , & Salas‐Mellado, M. (2014). Chemical characterization of chia (*Salvia hispanica* L.) for use in food products. Journal of Food and Nutrition Research, 2(5), 263–269. 10.12691/jfnr-2-5-9

[fsn34200-bib-0018] Coorey, R. , Tjoe, A. , & Jayasena, V. (2014). Gelling properties of chia seed and flour. Journal of Food Science, 79(5), E859–E866. 10.1111/1750-3841.12444 24734892

[fsn34200-bib-0019] Cuomo, F. , Iacovino, S. , Cinelli, G. , Messia, M. C. , Marconi, E. , & Lopez, F. (2020). Effect of additives on chia mucilage suspensions: A rheological approach. Food Hydrocolloids, 109, 106118. 10.1016/j.foodhyd.2020.106118

[fsn34200-bib-0020] da Silveira Ramos, I. F. , Magalhães, L. M. , do O Pessoa, C. , Pinheiro Ferreira, P. M. , dos Santos Rizzo, M. , Osajima, J. A. , Silva‐Filho, E. C. , Nunes, C. , Raposo, F. , Coimbra, M. A. , Ribeiro, A. B. , & Costa, M. P. (2021). New properties of chia seed mucilage (*Salvia hispanica* L.) and potential application in cosmetic and pharmaceutical products. Industrial Crops and Products, 171, 113981. 10.1016/j.indcrop.2021.113981

[fsn34200-bib-0021] Dai, S. , Jiang, F. , Shah, N. P. , & Corke, H. (2017). Stability and phase behavior of konjac glucomannan‐milk systems. Food Hydrocolloids, 73, 30–40. 10.1016/j.foodhyd.2017.06.025

[fsn34200-bib-0022] Daraghmah, F. S. , & Qubbaj, T. (2022). Impact of gum arabic and cactus mucilage as potential coating substances combined with calcium chloride treatment on tomato (*Solanum lycopersicum* L.) fruit quality attributes under ambient storage conditions. Canadian Journal of Plant Science, 102, 375–384. 10.1139/cjps-2021-0164

[fsn34200-bib-0023] Doğan, M. , & Kayacier, A. (2004). Rheological properties of reconstituted hot salep beverage. International Journal of Food Properties, 7(3), 683–691. 10.1081/JFP-200033093

[fsn34200-bib-0024] Feizi, R. , Goh, K. K. T. , & Mutukumira, A. N. (2021). Effect of chia seed mucilage as stabiliser in ice cream. International Dairy Journal, 120, 105087. 10.1016/j.idairyj.2021.105087

[fsn34200-bib-0025] Fernandes, S. S. , & Salas‐Mellado, M. M. (2017). Development of mayonnaise with substitution of oil or egg yolk by the addition of chia (*Salvia hispanica* L.) mucilage. Journal of Food Science, 83(1), 74–83. 10.1111/1750-3841.13984 29165817

[fsn34200-bib-0026] Garcia e Silva, L. L. , Bastos, R. A. , Lima, G. V. S. , Soares, L. S. , Coimbra, J. S. R. , Martins, M. A. , & Santana, R. C. (2022). Stabilizing properties of chia seed mucilage on dispersions and emulsions at different pHs. Food Biophysics, 17, 568–574. 10.1007/s11483-022-09742-x

[fsn34200-bib-0027] Georgiadis, N. , Ritzoulis, C. , Charchari, E. , Koukiotis, C. , Tsioptsias, C. , & Vasiliadou, C. (2012). Isolation, characterization and emulsion stabilizing properties of polysaccharides form orchid roots (salep). Food Hydrocolloids, 28, 68–74. 10.1016/j.foodhyd.2011.12.001

[fsn34200-bib-0028] Gürsel, A. , & Anlı, E. A. (2018). İçme Sütü Analiz Yöntemleri. In Z. Öner & H. Şanlıdere Aloğlu (Eds.), Süt ve Süt Ürünleri Analiz Yöntemleri (pp. 129–131). Sidas Medya.

[fsn34200-bib-0029] Gürsoy, O. , Kocatürk, K. , Güler Dal, H. Ö. , Yakalı, H. N. , & Yılmaz, Y. (2020). Physicochemical and rheological properties of commercial kefir drinks. Akademik Gıda, 18(4), 375–381. 10.24323/akademik-gida.850881

[fsn34200-bib-0030] Gürsoy, O. , Yılmaz, Y. , Gökçe, Ö. , & Ertan, K. (2016). Effect of ultrasound power on physicochemical and rheological properties of yoghurt drink produced with thermosonicated milk. Emirates Journal of Food and Agriculture, 28(4), 235–241. 10.9755/ejfa.2015-09-719

[fsn34200-bib-0031] Kayacier, A. , & Doğan, M. (2006). Rheological properties of some gums‐salep mixed solutions. Journal of Food Engineering, 72, 261–265. 10.1016/j.jfoodeng.2004.12.005

[fsn34200-bib-0032] Koocheki, A. , Taherian, A. R. , & Boston, A. (2013). Studies on the steady shear flow behavior and functional properties of *Lepidium perfoliatum* seed gum. Food Research International, 50, 446–456. 10.1016/j.foodres.2011.05.002

[fsn34200-bib-0033] Koocheki, A. , Taherian, A. R. , Razavi, S. M. A. , & Bostan, A. (2009). Response surface methodology for optimization of extraction yield, viscosity, hue and emulsion stability of mucilage extracted from *Lepidium perfoliatum* seeds. Food Hydrocolloids, 23, 2369–2379. 10.1016/j.foodhyd.2009.06.014

[fsn34200-bib-0034] Kreutz, C. A. J. (2002). Türkiye'nin orkideleri, salep, dondurma ve katliam. Yeşil Atlas, 5, 98–109.

[fsn34200-bib-0035] Kurt, A. , & Atalar, İ. (2018). Effects of quince seed on the rheological, structural and sensory characteristics of ice cream. Food Hydrocolloids, 82, 186–195. 10.1016/j.foodhyd.2018.04.011

[fsn34200-bib-0036] Liu, Q. , Qi, J. , Yin, S. , Wang, J. , Guo, J. , Feng, L. , Cheng, M. , Cao, J. , & Yang, X. (2017). Preparation and stabilizing behavior of octenyl succinic esters of soybean soluble polysaccharide in acidified milk beverages. Food Hydrocolloids, 63, 421–428. 10.1016/j.foodhyd.2016.09.020

[fsn34200-bib-0037] McLellan, M. R. , Lind, L. R. , & Kime, R. W. (1995). Hue angle determinations and statistical analysis for multiquadrant hunter L, a, b data. Journal of Food Quality, 18, 235–240. 10.1111/j.1745-4557.1995.tb00377.x

[fsn34200-bib-0038] Muñoz, L. A. , Cobos, A. , Diaz, O. , & Aguilera, J. M. (2012). Chia seeds: Microstructure, mucilage extraction and hydration. Journal of Food Engineering, 108, 216–224. 10.1016/j.jfoodeng.2011.06.037

[fsn34200-bib-0039] Sandoval‐Oliveros, M. R. , & Paredes‐Lopez, O. (2013). Isolation and characterization of proteins from chia seeds (*Salvia hispanica* L.). Journal of Agriculture and Food Chemistry, 61, 193–201. 10.1021/jf3034978 23240604

[fsn34200-bib-0040] Şen, M. A. (2016). Türkiye'nin Değişik Yörelerinden Toplanan Orkidelerden Elde Edilen Saleplerin Özelliklerinin Belirlenmesi ve Geleneksel Yöntemle Maraş Usulü Dondurma Yapımında Ürün Kalitesine Etkilerinin Araştırılması, Ph.D. Thesis. Namık Kemal University Graduate School of Natural and Applied Sciences, Tekirdağ, Turkey.

[fsn34200-bib-0041] Şen, M. A. (2017). Topraktan külaha yasak hazine “salep”. Gıda Mühendisliği Dergisi, 42, 65–69.

[fsn34200-bib-0042] Sezik, E. (2002). Turkish orchids and salep. Acta Pharmaceutica Turcica, 44, 151–157.

[fsn34200-bib-0043] Steffe, J. F. (1996). Rheological Methods in food process engineering (2nd ed.). Freeman Press.

[fsn34200-bib-0044] Szczesniak, A. S. , & Farkas, E. (1962). Objective characterization of the mouthfeel of gum solutions. Journal of Food Science, 27, 381–385. 10.1111/j.1365-2621.1962.tb00112.x

[fsn34200-bib-0045] Tamer, C. E. , Karaman, B. , & Copur, O. U. (2006). A traditional Turkish beverage: Salep. Food Reviews International, 22(1), 43–50. 10.1080/87559120500379902

[fsn34200-bib-0201] Tas, E. B. , Dundar, F. , Ozgur, G. Yilmaz, Y. & Gursoy, O. (2023). Effect of chia (Salvia hispanica L.) seed mucilage powder on some physicochemical and rheological properties of ayran drinks. Mljekarstvo, 73(2), 118–125. 10.15567/mljekarstvo.2023.0205

[fsn34200-bib-0046] Tekinşen, K. K. , & Güner, A. (2010). Chemical composition and physicochemical properties of tubera salep produced from some *Orchidaceae* species. Food Chemistry, 121, 468–471. 10.1016/j.foodchem.2009.12.066

[fsn34200-bib-0047] Timilsena, Y. P. , Adhikari, R. , Kasapis, S. , & Adhikari, B. (2016). Molecular and functional characteristics of purified gum from Australian chia seeds. Carbohydrate Polymers, 136, 128–136. 10.1016/j.carbpol.2015.09.035 26572338

[fsn34200-bib-0048] Yaşar, K. , Kahyaoğlu, T. , & Şahan, N. (2009). Dynamic rheological characterization of salep glucomannan/galactomannan based milk beverages. Food Hydrocolloids, 23, 1305–1311. 10.1016/j.foodhyd.2008.11.005

